# Rapid Chromatographic and Spectroscopic Analysis of Extracted Raw Propolis

**DOI:** 10.3390/molecules30244729

**Published:** 2025-12-10

**Authors:** Darinka Cvetković, Maja Somogyi Škoc, Ernest Meštrović, Iva Rezić Meštrović

**Affiliations:** 1 Department of Materials, Fibers and Textile Testing, Faculty of Textile Technology, University of Zagreb, 10000 Zagreb, Croatia; 2 Department of General and Inorganic Chemistry, Faculty of Chemical Engineering, University of Zagreb, 10000 Zagreb, Croatia; emestrov@fkit.unizg.hr; 3 Department of Applied Chemistry, Faculty of Textile Technology, University of Zagreb, 10000 Zagreb, Croatia

**Keywords:** propolis, thin layer chromatography, FTIR, UV-VIS, coffee ring

## Abstract

Propolis is a complex mixture of natural compounds, including resinous terpenoids, flavonoids, aromatic acids, and essential oils, and has strong antimicrobial, antifungal, and antioxidant properties. The chemical composition of propolis determines its properties and strongly depends on a wide variety of different plant sources, as well as other climate and environmental parameters. In order to determine the main compounds, in this study, we applied an integrated analysis of propolis by thin-layer chromatography (TLC) to characterize and compare the phytochemical profiles of selected bioactive materials in raw propolis. TLC served as a rapid, cost-effective, and highly visual technique to separate and identify key constituents, including terpenoids, flavonoids, and phenolic compounds in propolis, without a need for further precleaning steps after performing ultrasonic extraction. Complementary methods, such as FTIR spectroscopy, were employed to validate and quantify the active components detected through TLC screening. In addition, the UV-VIS method revealed the solubility of raw propolis in different solvents, after testing for coffee ring effects. The results confirmed that the complex structure of the raw sample can be more thoroughly revealed by two-dimensional TLC, which enables not only rapid and verifiable qualitative results but also detection of overlapping spots. Moreover, by comparing the results with data from the literature, not only can particular chemical compounds be efficiently determined by TLC but also the regional origin of samples.

## 1. Introduction

**Figure 1:**
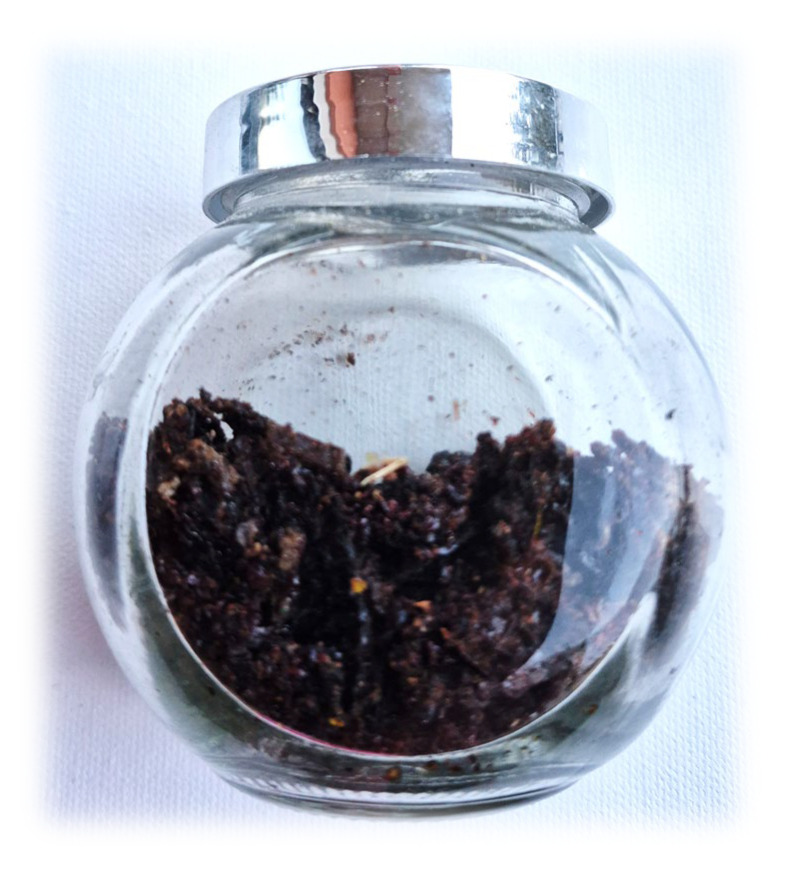
Raw propolis sampled directly from a bee hive in Zagreb, Croatia, dating October 2025.

Propolis is a chemically complex natural substance composed of a wide array of bioactive compounds, making its separation and comprehensive analysis a considerable analytical challenge [1,2,3]. Flavonoids, aromatic acids, phenolic acids, and their esters are key bioactive compounds in propolis, contributing to their diverse pharmacological properties, such as antioxidant, antibacterial, antiparasitic, antiviral, antileishmanial, antidiabetic, anti-inflammatory, immunomodulatory, and anticancer effects [4,5]. As such, propolis stands out as an extraordinary natural product with a broad spectrum of biological activities originating from its chemical composition [1,6,7,8]. Propolis is a resinous mixture collected by honeybees from plant exudates, enriched with beeswax and salivary enzymes. The composition of raw propolis (Figure 1) varies by geographic region and plant source but commonly includes flavonoids, phenolic acids, terpenoids, and aromatic aldehydes.

Propolis has been known for its potent antimicrobial, antiviral, antifungal, and anti-inflammatory properties [1]. Propolis has been used for centuries in traditional medicine to treat wounds, infections, and respiratory ailments [2]. It acts by inhibiting microbial adhesion, disrupting cell walls, and modulating inflammatory responses. Clinical and in vitro studies show strong effects against *Staphylococcus aureus*, *Candida albicans*, herpes simplex virus, and influenza viruses [4]. Propolis is also used in oral care products, cosmetics, dietary supplements, and topical ointments, as its synergistic bioactivity makes it one of the most effective bee-derived natural medicines.

Natural resins, including propolis, have long been used in traditional medicine across diverse cultures due to their wide range of biological activities. Secreted by trees and shrubs as a defense against pathogens and injury, these complex mixtures contain numerous bioactive constituents such as terpenoids, flavonoids, phenolic acids, and essential oils [1,2,3,4,5]. Recent research has intensified around their antimicrobial and antiviral potential, driven by growing antimicrobial resistance and the search for alternative therapeutics. Many in vitro studies report that specific resins strongly inhibit both Gram-positive and Gram-negative bacteria, as well as fungal strains [4]. However, their complex phytochemical nature complicates standardization and reproducibility, as composition varies by botanical and geographical source. Advanced chromatographic methods, including TLC profiling, are essential for identifying active compounds and ensuring quality control [5]. As global interest in natural therapeutics grows, further pharmacological and clinical research is needed to validate their efficacy. Natural resins remain a promising yet underexplored source of bioactive molecules with potential to inspire next-generation anti-infective and anti-inflammatory drugs.

Thin-layer chromatography (TLC) is a simple, rapid, and widely used method for qualitative analysis. Propolis compounds can be easily tested by TLC, which enables not only chemical analysis but also a regional detection of the sample origin, since thin-layer chromatography (TLC) offers a practical and informative method for the preliminary assessment of propolis authenticity and quality, particularly through its phytochemical fingerprint [1,2,3,9]. Chemical profiles of propolis vary substantially and are shaped by several factors, including geographical location, the botanical sources available to bees, and the specific bee species involved in its production. The chemical composition of propolis typically includes approximately 50% plant resins, 30% waxes, 10% essential oils, 5% pollen, and 5% other organic substances such as sugars and amino acids. Propolis from temperate regions is characterized mainly by flavonoids lacking B-ring substituents, including chrysin, galangin, pinocembrin, and pinobanksin. Caffeic acid phenethyl ester (CAPE), a major constituent of temperate propolis, exhibits broad biological activities, such as inhibition of nuclear factor κB, suppression of cell proliferation, and induction of cell cycle arrest and apoptosis. In contrast, tropical propolis—particularly Brazilian green propolis—is dominated by prenylated phenylpropanoids (e.g., artepillin C) and diterpenes. Propolis from the Pacific region is distinguished by geranyl flavanones, compounds that are also present in certain African propolis types [10].

**Figure 2:**
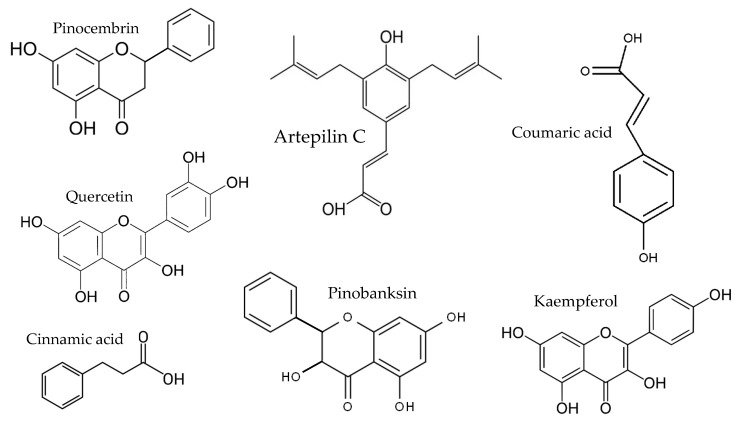
Chemical components monitored by TLC in propolis samples.

In temperate regions such as Serbia, Slovenia, and Croatia, propolis is predominantly derived from exudates of Populus species. These are rich in phenolic constituents, including flavones, flavanones, phenolic acids, and their corresponding esters [11,12,13,14,15,16,17,18,19]. Characteristic compounds commonly identified in chromatograms of poplar-type propolis include galangin, chrysin, naringenin, ferulic acid, caffeic acid, myricetin, gallic acid, and chlorogenic acid (Table 1), as well as artepilin C, cinnamic acid, coumaric acid, kaempferol, pinobanksin, pinocembrin, and quercetin (Figure 2) [10,20,21,22].

In previous experiments, the use of alternative extraction methods and stationary phases further influenced band intensity and resolution, emphasizing the importance of analytical parameters in propolis profiling [23,24,25]. While TLC provides a reliable preliminary tool for assessing phenolic composition and differentiating chemotypes, complementary quantitative and spectroscopic analyses are required to identify definitive quality markers essential for propolis classification and standardization. In this work, two-dimensional TLC will be used for verification of results of 1D TLC.

Two-dimensional thin-layer chromatography (2D-TLC) offers a significant improvement over traditional one-dimensional TLC by providing enhanced separation, resolution, and identification of complex mixtures. In 1D-TLC, components may overlap when they possess similar polarity or migration behaviors, limiting accurate interpretation. In contrast, 2D-TLC involves developing the same plate sequentially in two perpendicular directions using different solvent systems, allowing compounds that co-migrate in the first dimension to be separated in the second. This orthogonal approach provides a higher degree of selectivity and separation power, making 2D-TLC especially valuable for analyzing natural extracts, such as propolis, that contain numerous chemically similar compounds [1,9].

The coffee ring effect is a phenomenon in which particles suspended in a liquid droplet migrate toward the edges during evaporation, forming a ring-like deposit due to capillary flows that compensate for faster edge evaporation. This effect can be examined by preparing a homogeneous solution of the substance of interest (e.g., propolis extract), depositing a small droplet (2–5 μL) on a clean substrate such as glass, silicon, or a TLC plate, and allowing it to dry under controlled conditions. The resulting deposits are analyzed using optical microscopy or imaging to assess particle distribution, particularly edge accumulation, and can be further characterized with spectroscopic or chromatographic techniques like FTIR [12,13,14,15], UV-VIS [10,11], or TLC [1,2,3]. This approach offers a simple, non-destructive means to study particle–solvent interactions, solubility, viscosity, stability, and component uniformity. When applied to propolis analysis, the coffee ring effect serves as a powerful complement to traditional chromatography, enhancing the resolution and interpretability of TLC results. Observing the spatial distribution of compounds such as phenolic acids, flavonoids, and resins provides valuable insight into aggregation behavior, chemical heterogeneity, and component interactions, ultimately improving the analytical characterization and formulation optimization of propolis extracts.

In this work we investigated a simple, rapid, inexpensive, sensitive, and specific method for the identification of compounds in propolis extract samples with the main aim being to describe a rapid method for analysis of raw propolis samples sampled directly from a bee hive, without additional purification except from proposed ultrasonic extraction, UAE, by using (HP)TLC [(high-performance) thin-layer chromatography] combined with videodensitometric post-chromatographic detection under visible and UV light. For the first time, to our knowledge, we propose in this work a chromatographic system with benzene-MeOH (95:5, *v*/*v*) for the analysis of propolis samples and compare the obtained results to the results of other antimicrobial, antiviral, anti-inflammatory natural compounds. In addition, two-dimensional TLC was performed for validation of the results, and FTIR and UV-VIS spectroscopic analysis were used to further characterize natural propolis samples.

## 2. Results and Discussion

**Figure 3:**
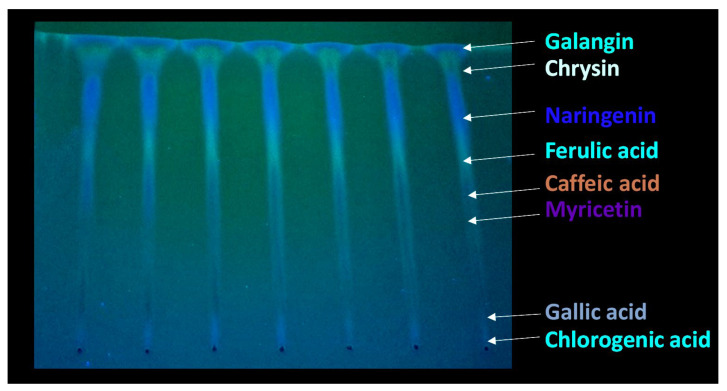
Chromatographic plate Silicagel F254s, recorded under UV light of 366 nm, after developing propolis extract sample (1 g in 3 mL MeOH after 10 min of UAE); 7 repeated samples with detected compounds of chlorogenic acid, gallic acid, myricetin, caffeic acid, ferulic acid, naringenin, chrysin, and galangin, a selection recorded under UV lamp.

**Figure 4:**
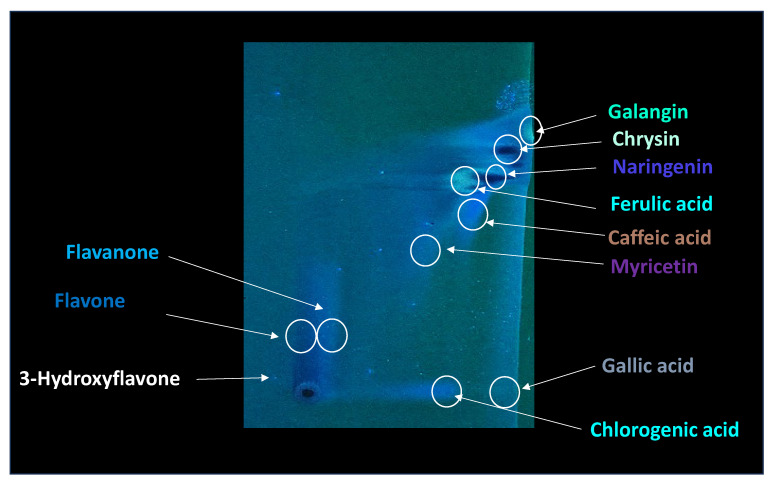
Recorded results of two-dimensional TLC of propolis extracts, developer benzene-MeOH (19-1), Silicagel F254s, recorded under UV light of 366 nm under UV lamp. Sample preparation: A total of 1 g of pure solid propolis extracted in 3 mL of MeOH for 10 min by sonication without heating. Components detected: galangin, chrysin, naringenin, ferulic acid, caffeic acid, myricetin, flavanone *, flavone *, 3-hydroxyflavone *, and separated gallic acid from chlorogenic acid. * Flavanone, flavone, and 3-hydroxyflavone were not visible in 1D TLC.

TLC results of a propolis sample are shown in Figure 3 and Figure 4. Figure 3 presents the chromatographic plate Silicagel F254s, recorded under UV light of 366 nm, after developing a propolis extract sample, with 7 repeated samples used for determination of repeatability. Propolis compounds determined through their R_F_ values are listed in Table 1.

In order to detect particular compounds of interest, two-dimensional TLC was performed, and the results are presented in Figure 4.

**Figure 5:**
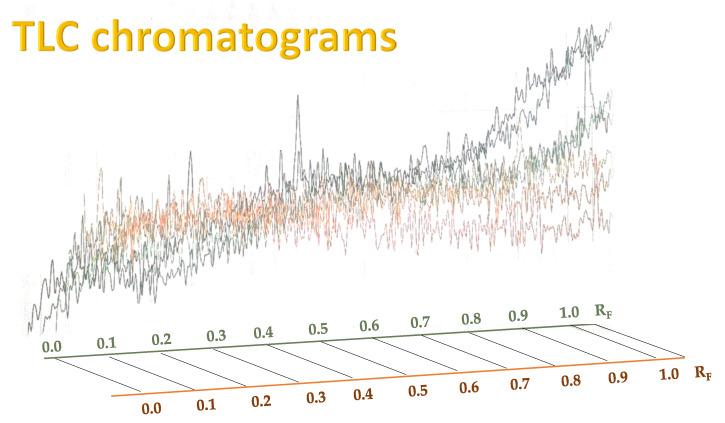
Figure of TLC chromatograms of one-dimensional TLC produced by VisionCATS 2.0 software after recording the chromatographic plate Silicagel F254s under UV light of 366 nm of propolis extract sample (1 g in 3 mL MeOH after 10 min of UAE), 7 repeated samples with detected compounds including chlorogenic acid, gallic acid, myricetin, caffeic acid, ferulic acid, naringenin, chrysin, and galangin.

As can be seen from Figure 3 and Figure 4, as well as in Table 1, some of the compounds in propolis extract had very similar chemical compositions, which resulted in similar chemical behavior, resulting in very closely positioned spots with similar R_F_ values; due to this, their separation in 1DTLC was not possible, not even after using a very long path for development of 18 cm instead of the usual 9 cm. For example, the position of the compound 3-Hydroxyflavone with an R_F_ value of 0.13 was very close to the position of gallic acid, which had R_F_ 0.08, and due to broadening of their spots, there was overlap, resulting in significantly poor resolution for detection of 3-Hydroxyflavone. Similar problems occurred for flavone (R_F_ 0.44) and flavanone (R_F_ 0.50), which were not separated nor detected in the presence of myricetin (R_F_ 0.54) by using 1D TLC. In contrast, all of those compounds are visible on the chromatographic plate after 2D TLC separation. This can be further confirmed by visual inspection of the obtained chromatograms (Figure 5). Recent advancements in digital scanning of TLC chromatograms have enabled visual inspection of chromatograms, providing valuable information not only on the R_F_ values of particular components but also on their quantity in the sample. Figure 5 shows the TLC chromatograms of corresponding propolis samples, recorded on a plate shown in Figure 3.

As can be seen from Figure 5, the chromatogram of the raw propolis extract sample, analyzed seven times, shows that the sample has many compounds which are hard to distinguish. In contrast, when identifying compounds by their colors and R_F_ values and data from the literature, the most prominent species can be identified (Figure 3 and Figure 4, Table 1). The identification is much easier with two-dimensional chromatogram, as presented in Figure 4. When comparing results presented in Figure 4 (two-dimensional TLC) and Figure 5 (one-dimensional TLC coupled with videoscanning), it can be concluded that, although two-dimensional thin-layer chromatography (2D-TLC) represents an advanced analytical approach that provides superior qualitative identification of compounds, particularly in complex natural samples such as propolis, videoscanning enables quantification of particular sample compounds present in one track from the one-dimensional TLC.

The principle of 2D-TLC lies in sequential development of the same chromatographic plate in two directions using different solvent systems, each with distinct selectivity and polarity. This dual development significantly improves the resolution of overlapping spots that often occur in one-dimensional (1D) TLC. Since propolis contains a wide range of phenolic acids, flavonoids, and terpenoids with varying polarities, 2D-TLC enables a more comprehensive chemical fingerprint by minimizing co-elution and enhancing spot separation. Compounds that may appear as a single streak in a 1D-TLC run can be distinctly resolved into separate zones after the second development, allowing a more accurate qualitative profile. In particular, 2D-TLC helps distinguish structurally similar compounds such as gallic acid and chlorogenic acid or flavonoids with close polarity values such as naringenin, chrysin, and galangin. The improved selectivity achieved through the orthogonal solvent systems in 2D-TLC offers deeper insight into the compositional complexity of propolis and supports chemotaxonomic classification and authentication studies.

However, despite its superior qualitative resolving power, 2D-TLC poses significant limitations in quantitative analysis due to the complexity of spot geometry and overlapping in two directions, which make precise densitometric scanning difficult. In contrast, one-dimensional TLC (1D-TLC) provides a more practical and quantifiable analytical format. Using a single solvent system for linear development, 1D-TLC plates can be scanned under a video densitometer to obtain both qualitative and quantitative data. The CAMAG videodensitometer (CAMAG, Bonaduz, Switzerland) combined with VisionCATS software (CAMAG, Bonaduz, Switzerland), for instance, allows high-resolution image capture of the chromatographic plate under ultraviolet light (typically at λ = 254 nm) and enables digital evaluation of the intensity, area, and position of each separated spot. This provides accurate relative concentration data for individual compounds while maintaining qualitative identification through retention factor (R_F_) values and UV fluorescence profiles. In the analysis of propolis extracts, VisionCATS software can effectively detect and quantify bioactive constituents such as galangin, chrysin, naringenin, ferulic acid, caffeic acid, myricetin, flavanone, flavone, and 3-hydroxyflavone, as well as differentiate closely eluting compounds like gallic acid and chlorogenic acid. The software’s integrated database and comparison tools further enhance identification accuracy by referencing chromatographic patterns of standards alongside the test sample.

Thus, while 2D-TLC offers an unparalleled ability to qualitatively resolve and identify a broader range of compounds in propolis—making it invaluable for exploratory and comparative phytochemical profiling—it lacks the straightforward quantitative interpretability that 1D-TLC provides. The single-dimensional format ensures reproducible migration paths, enabling the densitometric quantification of individual analytes with high accuracy and minimal interference. Consequently, an optimal analytical workflow often combines the strengths of both methods: 2D-TLC is first employed for comprehensive qualitative mapping and identification of overlapping or unknown compounds, followed by targeted 1D-TLC with videodensitometric analysis for quantitative determination of key bioactive markers. This complementary approach ensures that both the qualitative complexity and quantitative precision of propolis composition are fully characterized, providing a robust foundation for quality control, standardization, and pharmacological evaluation of this biologically active natural material.

The coffee ring effect represents a fundamental self-organization process that occurs during solvent evaporation from a droplet containing suspended solutes or particles. As evaporation proceeds, capillary flows drive the suspended matter from the center toward the periphery, producing a characteristic ring-shaped deposit. This microscale mass transport phenomenon, long regarded as an artifact in drying studies, has gained growing attention for its relevance to particle dynamics, solvent–solute interactions, and micro-pattern formation [26]. In analytical chemistry, understanding and controlling the coffee ring effect is increasingly recognized as a means to probe physicochemical properties such as solubility, viscosity, and colloidal stability, offering a visual analog to chromatographic separation processes.

Recent advances in digital microscopy, optical profilometry, and image-based quantification have enabled precise characterization of coffee ring morphology and spatial solute distribution [27,28,29,30]. By modulating parameters such as solvent composition, droplet volume, substrate surface energy, and evaporation rate, researchers can now tailor deposition patterns to investigate aggregation behavior, particle–matrix affinity, and the mobility of bioactive molecules [28]. Controlled droplet drying, particularly in studies of natural extracts like propolis or plant resins, provides rapid insights into solute heterogeneity and potential interactions among complex mixtures—analogous to chromatographic migration on thin-layer plates [31,32,33].

In this context, the coffee ring effect may be regarded as a two-dimensional spontaneous chromatographic phenomenon, governed by radial convective flows rather than capillary-driven solvent fronts. While TLC achieves spatial resolution through controlled solvent migration along a coated adsorbent layer, the coffee ring effect offers a complementary evaporative pathway to visualize compound mobility and segregation. Both approaches share the underlying principle of differential solute transport and surface adsorption. When examined in conjunction, they provide a comprehensive perspective on material behavior—TLC offering analytical separation and identification, and the coffee ring effect revealing physicochemical gradients and aggregation dynamics at the microscale.

Integrating coffee ring analysis with thin-layer chromatography thus opens new possibilities for material characterization and natural product research. For example, analyzing a dried droplet of propolis extract on a glass substrate can reveal concentration-dependent edge effects and aggregation tendencies, while corresponding TLC profiles provide precise chemical identification of the separated constituents. The synergy between these two methods establishes a bridge between physical and chemical analysis—where the visual, spatial distribution seen in coffee ring patterns complements the molecular resolution achieved through TLC. In essence, just as seeding controls crystal growth in pharmaceutical crystallization, controlling evaporation and deposition in coffee ring formation guides the visualization of molecular interactions, linking droplet dynamics to chromatographic science in a unified analytical framework.

Despite the problems of poor detection of many components in one-dimensional TLC, chromatographic analysis of extracts of raw propolis compounds can provide a preliminary chemical profile or ‘fingerprint’ of a sample, which can be used as a path for determination of regional origin. Although the TLC by itself does not provide reliable evidence of a sample’s origin or authenticity, reliable determination of authenticity and geographical origin is possible by combination of TLC with other advanced analytical techniques. For example, Milojković Opsenica et al. have reported regional origin of propolis based on differences in obtained TLC chromatograms and principal component analysis [34]. They presented HPTLC chromatograms of propolis test solutions and corresponding standards from samples of known geographical and botanical origin (namely: Serbia, Croatia, Slovenia, Brazil, and France) and argued that, when individual compounds are considered, thin-layer chromatographic profiles can serve as useful indicators of propolis authenticity and quality. Serbia, Slovenia, and Croatia were grouped as temperate regions where propolis is predominantly derived from *Populus* species, whose exudates are rich in phenolic constituents, including flavones, flavanones, phenolic acids, and their esters [35,36]. Our results support their findings (Table 1, Figure 3) that chromatograms of the test samples showed that the numerous less-polar constituents—such as hydroxycinnamic acids, flavanols, flavanonols, and flavones—displayed higher R_F_ values. The TLC profiles revealed clear differences in the phytochemical makeup of the analyzed extracts and supported the presence of two principal poplar propolis types. Samples classified as the orange type exhibited several intense orange zones accompanied by a few pale blue and faint green ones. In contrast, the blue type was distinguished by prominent dark and light blue zones, along with weaker orange and light green bands. The orange-type chromatograms appeared relatively uniform, whereas those of the blue-type propolis displayed more variable patterns and generally lower zone intensities, suggesting a reduced phenolic content. Therefore, a visual inspection of the chromatograms demonstrates clear chemical differences among samples from different regions, which leads to the conclusion that Serbian propolis test solutions largely matched the orange-type profile, while Croatian samples also aligned with the orange type but exhibited less intense bands, while Slovenian chromatograms more closely resembled those typical of blue-type propolis [34].

Because propolis composition is strongly influenced by its botanical source—which is itself shaped by the geographical environment—samples originating from the same plant species may still vary chemically. Such differences arise from variations in climate (e.g., sunlight and humidity), soil properties, and mineral content, all of which affect plant secondary metabolite accumulation. The study demonstrated that chemical profiles of propolis from the same botanical source can indeed be linked to their geographical origin. However, the authors emphasized that more advanced analytical techniques, such as high performance liquid chromatography (HPLC) coupled with mass spectrometry, are required for deeper and more precise characterization [37,38]. Without better detection, the differentiation among propolis compounds is almost impossible due to many competing species within the sample. Medić Šarić et al. reported R_F_ reference values for flavonoids and phenolic acids which are present in propolis, which were obtained from nine different chromatographic systems [39]. Differentiation among the systems was compared based on a selection of a few of the investigated systems and is shown in Table 2.

As can be seen from Table 2, many propolis compounds overlap and strongly correlate to the polarity of solvents used as a mobile phase, and the situation worsens in the raw propolis extract samples which were analyzed without additional pre-cleaning steps (Figure 5), after proposed ultrasonic extraction in MeOH. Therefore, further optimization of the chromatographic TLC system is required for detection of particular components of interest. Moreover, it has to be emphasized that the optimal solvent in this study required benzene, which should be replaced with less toxic compounds for further optimization.

**Figure 6:**
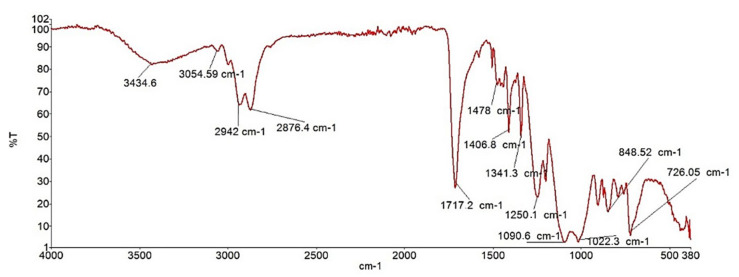
FTIR spectrum of propolis sample.

In this work, the raw propolis extract sample was further investigated by FTIR and UV-VIS spectroscopy. Firstly, FTIR was recorded and the result is presented in Figure 6.

As shown in Figure 5, the FTIR spectrum displays a distinct O–H stretching band characteristic of phenolic compounds at 3425 cm^−1^, while in the fingerprint region, bending vibrations of CH_2_ and O–H appear at 727 cm^−1^, also typical of phenols and hydrocarbons. Several characteristic frequencies for aromatic compounds are also evident, such as C–H stretching detected at 2922 cm^−1^. As a result of aromatic ring stretching, sharp peaks at 1458 cm^−1^ and 1582 cm^−1^ correspond to C–C bond stretching vibrations within aromatic structures.

The FTIR spectrum also enables the detection of esters in the sample of propolis [40]. A strong stretching signal at 1718 cm^−1^ indicates the presence of a carbonyl (C=O) group. Due to the sp^2^-hybridized carbon, C–O bending vibrations appear at 1244 cm^−1^ in the fingerprint region, while a peak at 1096 cm^−1^ corresponds to C–O bending associated with sp^3^-hybridized carbon atoms.

Phenolic compounds, which are essential components of propolis, can also be readily identified using FTIR. The absorption bands of phenolic functional groups are observed as bending vibrations in the fingerprint region (1244 cm^−1^ and 830 cm^−1^), while ester functional groups appear at 980 cm^−1^ and 1098 cm^−1^. Flavonoid functional groups are detected at slightly higher frequencies (1458 cm^−1^, 1411 cm^−1^, and 1311 cm^−1^), with additional stretching vibrations around 1520 cm^−1^ typical of flavonoid structures. Bending vibration at 1411 cm^−1^ corresponds to dodecanol (dodecyl aldehyde). In the stretching region, a peak at 2858 cm^−1^ is characteristic of hydrocarbons.

**Figure 7:**
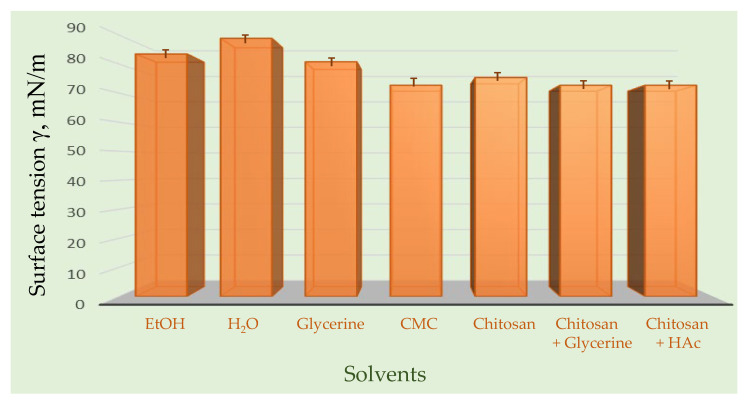
Comparison of surface tension of seven different solvent systems.

Surface tension of investigated samples in different solvents is presented in Figure 7.

Surface tension plays a fundamental role in both the coffee ring effect and thin-layer chromatography (TLC), linking these seemingly distinct phenomena through fluid dynamics and molecular interactions on solid surfaces. In the case of propolis, a complex natural mixture rich in flavonoids, phenolic acids, and terpenoids, the behavior of its extract during drying and chromatographic separation is strongly influenced by surface tension gradients. When a droplet of propolis extract dries on a glass or silica surface, the difference in surface tension between the edge and center of the droplet drives capillary flow, transporting solutes toward the perimeter—a process known as the coffee ring effect. This leads to visible ring-like deposits of phytochemicals, reflecting their differential solubility, volatility, and interaction with the surface. Similarly, in TLC, surface tension governs the capillary rise in the solvent through the stationary phase, determining the rate and uniformity of analyte migration. Variations in surface tension between solvents and propolis constituents affect their mobility, resulting in distinct chromatographic zones that correspond to compounds of varying polarity. Thus, surface tension acts as a unifying physical parameter in both phenomena, controlling molecular transport, deposition, and separation—key aspects for characterizing complex natural mixtures like propolis.

In order to avoid the “coffee ring effect” during application of the sample, it is essential to choose the right solvent, which can be a problem, as many efficient solvents are toxic and not environmentally friendly (e.g., benzene). However, only a few milliliters of solvents are needed for efficient TLC due to the microgram amounts of samples used in the system. In contrast, liquid chromatography operating under high pressure and automatization such as HPLC requires significantly higher amounts of solvent to run repeated analyses. Although in TLC, aerosol applicators and instruments such as, for example, Linomat 5 can significantly reduce the coffee ring effect, in this work, we did not apply it, since the raw propolis extract sample was analyzed without any other additional precleaning steps other than ultrasonic extraction, which is favorable for Linomat 5.

Therefore, firstly the stalagmometric method was applied for determination of the surface tension, as this simple but precise technique relies on comparing the number of drops formed by a liquid of known surface tension with the number of drops produced by the liquid under investigation. In practice, a calibrated stalagmometer is filled with the test liquid, and the drops are allowed to fall under the influence of gravity. By carefully counting the number of drops and using a reference liquid with a well-established surface tension, the surface tension of the test liquid can be calculated. This method is widely used due to its simplicity, reproducibility, and ability to provide reliable measurements for a variety of liquids, including aqueous solutions, organic solvents, and complex mixtures such as natural extracts. The accuracy of the measurement depends on controlling factors such as temperature, liquid density, and drop formation rate. By applying the stalagmometer technique, one can quantitatively assess interfacial properties, which are essential for understanding phenomena like wetting, adhesion, and emulsification in both laboratory and industrial applications.

Secondly, for investigation of solubility of propolis extracts in different solvents, UV-VIS spectra of dissolved propolis in different solvents were tested. According to the obtained results of the UV-VIS spectrophotometric absorbance values, ethanol (EtOH) proved to be the most effective solvent (100% = 0.7372), followed by water (68.3%), glycerin (56.3%), and finally carboxymethyl cellulose (CMC), which exhibited only 6.54% of the solubility compared to ethanol. However, the mixture of chitosan and glycerin demonstrated superior performance compared to all pure solvents, containing 64% more dissolved active compounds than the best pure solvent. When comparing the chitosan–glycerin mixture to other solvents, it stands out as the most efficient (100% = 1.1386), followed by ethanol (64.75%), water (44.19%), glycerin (36.43%), and CMC (4.24%). Nevertheless, it is important to note that only the sample in ethanol exhibited a distinct minor peak around 900 nm, indicating the presence of additional active substances unique to the alcoholic extract.

**Figure 8:**
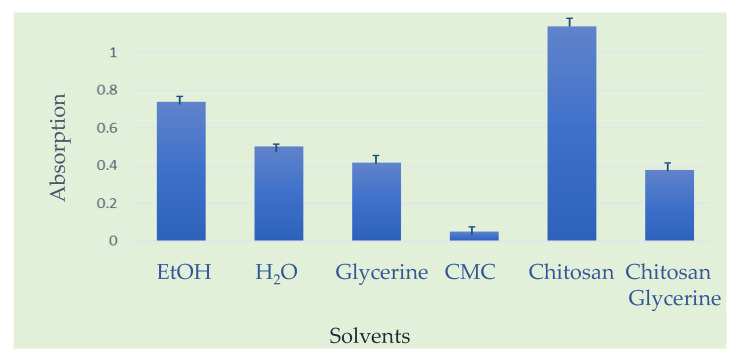
Solubility of propolis in different solvents (prior to TLC) monitored by UV-VIS spectrometry, Lambda 20, Perkin Elmer, Shelton, CT, USA.

A graphical presentation of solubility of propolis is presented in Figure 8.

In thin-layer chromatography, solvent strength and polarity directly influence the separation efficiency and resolution of complex mixtures such as propolis extracts. The mobile phase’s solvent system must be carefully chosen to balance interactions between the analytes and the stationary phase—typically silica gel, which is highly polar. A more polar solvent (for instance, methanol or ethyl acetate) increases the elution strength, allowing polar compounds like caffeic acid derivatives to migrate further up the TLC plate. Conversely, a less polar solvent (such as toluene or benzene) better separates nonpolar compounds like terpenoids by reducing their mobility and enhancing contrast between bands. In the case of propolis, using mixed solvent systems (e.g., benzene–methanol 95:5) provides optimal differentiation of its diverse chemical constituents by fine-tuning the solvent strength. Thus, understanding solvent polarity and elution strength is essential for achieving precise chromatographic separation, as these parameters govern how individual components interact with the adsorbent layer and move according to their polarity and affinity. Moreover, the strength and volatility of a solvent play a crucial role in determining the formation and morphology of the coffee ring effect. In a drying droplet of propolis extract, which contains compounds of varying polarity such as flavonoids, terpenoids, and phenolic acids, the solvent strength dictates how efficiently these components remain dispersed or migrate during evaporation. A strong, low-volatility solvent (such as ethanol or methanol) can maintain solute suspension longer, leading to a more uniform deposit and a weaker ring effect, while a weaker or more volatile solvent (like acetone or ethyl acetate) evaporates rapidly, enhancing the capillary flow that carries solutes toward the droplet edge. The balance between evaporation rate, surface tension, and solvent–solute interactions determines whether the final deposit appears as a distinct ring, a uniform film, or an irregular pattern. Therefore, solvent strength is a key factor in controlling how compounds from natural materials such as propolis are spatially distributed during drying, which has implications for studying deposition phenomena and film formation on various substrates.

**Figure 9:**
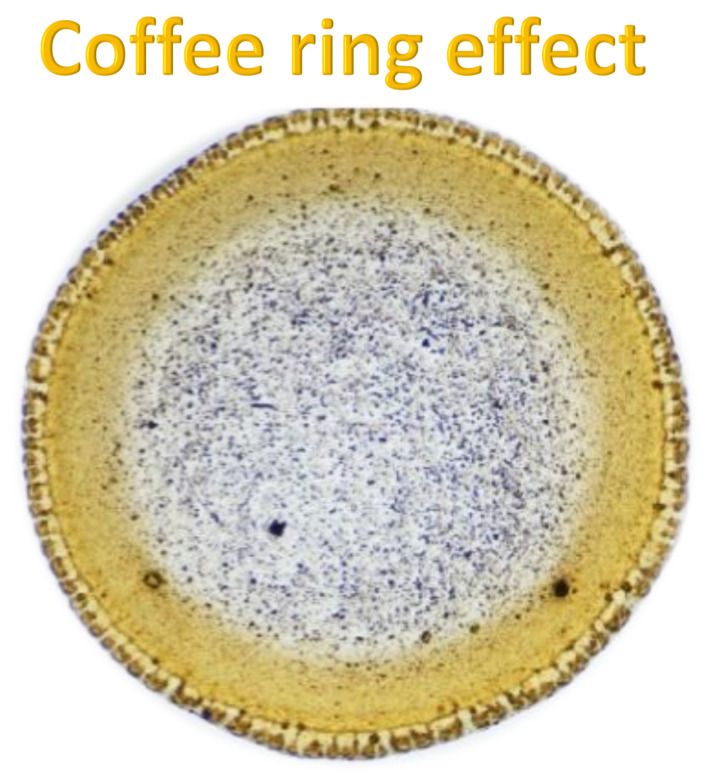
Microphotograph of coffee ring effect of selected active compound recorded by Olympus BX53M optical microscope.

Solvents influencing the coffee ring effects were obtained from droplets of solutions deposited on glass slides upon drying showed characteristic ring patterns that were formed, which allowed for comparison of morphological features and detection of changes caused by complexation (Figure 9).

The physical mixture of morin and the accompanying constituents, when dissolved in water, produced a distinctly granular coffee ring pattern with numerous visible crystals. This morphology is characteristic of systems with limited solubility and clearly indicates that complexation between the components did not occur to a meaningful extent. In contrast to the fully formed complex, the physical mixture displays noticeably lower homogeneity and a more irregular particle distribution across the dried droplet. These differences highlight how strongly the active components influence each other’s spatial arrangement within different solvent microenvironments.

This observation is particularly important for propolis analysis by TLC, where natural extracts contain a diverse array of flavonoids, phenolic acids, and other poorly soluble compounds. Understanding whether components interact as true complexes or only as loosely associated physical mixtures directly affects how they distribute during drying, how their morphology evolves, and how reliably they can be detected or quantified. The ability to distinguish granular, non-homogeneous patterns from uniform complex-driven structures therefore provides a valuable diagnostic tool for interpreting propolis composition, stability, and the behavior of its bioactive molecules in solution.

## 3. Materials and Methods

**Figure 10:**
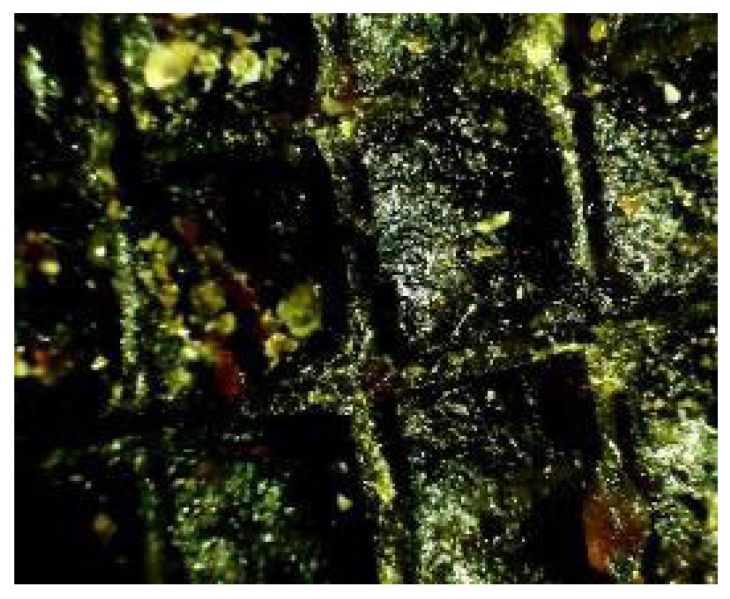
Microphotograph of propolis samples under magnification of 50× recorded after sampling it from bee hives.

Sampling of raw propolis was conducted directly from active honey bee (*Apis mellifera*) colonies to ensure collection of unaltered material representative of natural hive conditions. Propolis deposits were located along the inner margins of hive frames and entrance reducers, where resinous accumulations are commonly used by bees for structural reinforcement and antimicrobial defense. Using sterile stainless-steel spatulas, samples were carefully scraped to avoid contamination with wax, brood, or extraneous hive debris. All tools were disinfected with 70% ethanol between collections to prevent cross-hive microbial transfer. Immediately following removal, propolis samples were placed into pre-labeled sterile polypropylene tubes, stored in a cooled insulated container, and transported to the laboratory for subsequent physicochemical and microbiological analyses. This standardized direct-from-hive sampling procedure ensured the preservation of native chemical constituents and minimized post-collection alteration. Sampling was performed on Mount Medvednica, Zagreb County (Šestine), in Croatia, Europe by Darinka Cvetković, a Ph.D. student from the University of Zagreb, Department of Materials testing, Faculty of Textile Technology who is working under the supervision of Prof. Maja Somogyi Škoc (both are the coauthors of this investigation). Propolis sample shown in Figure 10, used in the research, was stored in a dry and dark place after sampling.

Approximately 1 g of raw propolis was suspended in 10 mL of MeOH and subjected to ultrasonic extraction for 15 min. The supernatant was collected, and the remaining solid residue was re-extracted under identical conditions. Extractions were performed at room temperature without additional heating; however, sonication-induced cavitation caused a slight temperature increase of a few degrees, reaching up to 30 °C. The two supernatants were combined and immediately analyzed by TLC. No evaporation or heating steps were applied to preserve the native chemical composition of the sample as much as possible. Consequently, the dry residue was not weighed, and the extract concentration was not determined.

**Figure 11:**
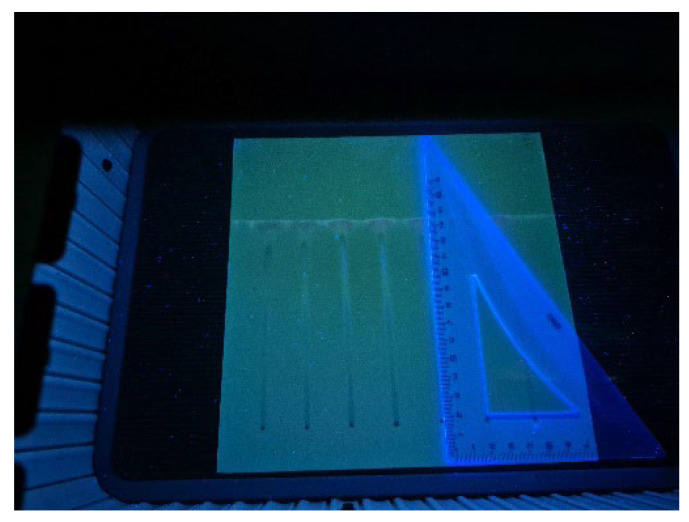
20 × 20 cm TLC Silicagel F254s plates (Merck, Darmstadt, Germany) with seven successive applications of extract of raw propolis samples after sonication, applied using a glass microcapillary positioned 20 mm among the spots, 20 mm from the lower edge and 15 mm from the left and the right plate borders, used in the identification of samples.

Aliquots of 2 μL of the resulting propolis extracts were applied to 20 × 20 cm TLC Silicagel F254s plates (Merck, Darmstadt, Germany) positioned minimally 10 to 20 mm from the lower edge and 15 to 20 mm from the left and the right plate borders, as well as among the spots (Figure 11). Application can be performed by using a Hamilton microsyringe (Bonaduz, Switzerland), but in case of the presence of undissolved particles in raw propolis, such materials can plug the syringe needle, resulting in unevenly applied spots, affecting reproducibility and chromatographic separation. The worst case scenario would be the malfunctioning of the Linomat automatic sampler and high costs for repairs. For this reason, in samples such as raw propolis, a better approach is to use glass microcapillaries for identification of unknown samples, making the quantification step not possible.

The use of 20 × 20 cm thin-layer chromatographic plates over 10 cm plates was primarily due to the increased resolution capabilities provided by the larger plate size. Additionally, larger plates can help in achieving better separation efficiency and reproducibility, as well as opening the possibility of handling samples with complex mixtures such as propolis.

Chromatographic separation was performed in a CAMAG chamber (Merck, Darmstadt, Germany) using the ascending technique to a distance of 18 cm, following 20 min of chamber saturation. Although TLC chromatograms are typically developed over a distance of approximately 9 cm, in the present study a longer development distance of 18 cm was employed to achieve improved separation. Raw propolis contains a complex mixture of numerous compounds, including flavonoids, phenolic acids, and their esters, which often co-migrate under standard conditions. According to established TLC theory, resolution generally increases with the length of the chromatographic path because longer migration distances allow for better differentiation of closely migrating components due to more effective partitioning between the stationary and mobile phases [32]. Therefore, extending the development distance provided enhanced resolution of the diverse constituents present in propolis, facilitating more accurate qualitative analysis (Figure 3).

The developing solvent system consisted of benzene–methanol (95:5, *v*/*v*), and for the two-dimensional TLC, the same solvent system was used.

Here, we report the rapid chromatographic system, in which the same solvent is used in both directions. As a further development, the second mobile phase can be completely different in composition, volume ratio, and visualization approach.

All reagents used in this research were of pro chromatography grade and obtained from the same company (Merck, Darmstadt, Germany). After the development, the plates were air-dried, and images were recorded under UV light at 254 nm and 366 nm, as well as under visible light, using the CAMAG VideoScan (CAMAG, Bonaduz, Switzerland) image analysis system coupled with a Reprostar 3 documentation unit (CAMAG, Bonaduz, Switzerland).

R_F_ value characterizes behavior in a specific chromatographic system, so if this was the only parameter of choice, there could be a high probability of error. Therefore, identification needed to be performed by comparing the retention of the substance in the sample with the retention of the reference substance in the same chromatographic system. In our case, due to easily distinguished colors, we decided, based on the literature, which components were to be placed in which position, and not by using R_F_ values from other chromatographic systems (Table 3). Table 3 presents different compositions of mobile phases that are efficient in analysis of propolis; the first mixture was used in this study and the others were tested in preliminary experiments.

Spectroscopic analysis included FTIR and UV-VIS spectrometry. FTIR analysis in the infrared region was performed using a Perkin Elmer Spectrum 100 FTIR spectrometer (Perkin Elmer, Shelton, CT, USA) equipped with an ATR accessory for surface analysis (FTIR-ATR). Spectra were recorded at room temperature over the range of 400–4000 cm^−1^.

UV–VIS analysis was carried out on a Perkin Elmer Lambda 20 spectrophotometer (Perkin Elmer, Shelton, CT, USA), a dual-beam instrument with a grating monochromator covering wavelengths from 190 to 1100 nm. The device uses halogen and deuterium lamps as light sources and a photodiode detector. Measurements were performed under the following conditions—wavelength range 200–1100 nm, slit width 2 nm, scan rate 240 nm/min, and 1.00 cm quartz cuvettes. Blank measurements were made using different solvents, depending on the extract type. The propolis extract showed a maximum absorption at λmax = 283.88 nm, consistent with data from the literature indicating flavonoid absorption between 250 and 400 nm, with characteristic peaks in the 250–290 nm range. After selecting the operational parameters, the UV-VIS methodology was used to determine the solubility of propolis in different solvents (prior to TLC). For this purpose, the same amount of raw propolis was added to different solvents or solvent mixtures, and quantification was performed by determination of the surface under the absorption curve obtained in the UV-VIS region on the characteristic chosen wavelengths.

The following solvent mixtures with propolis were applied: EtOH, water, chitosan (Sigma Aldrich, Burlington, MA, USA, CAS: 9012-76-4), and glicerin (C_3_H_8_O_3_, Grammol, Zagreb, Croatia, CAS: 56-81-5), as well as sodium carboxymethyl cellulose, [C_6_H_7_O_2_(OH)_2_(OCH_2_COONa)]_n_ (Sigma Aldrich, Burlington, MA, USA, CAS: 9004-32-4).

A total of 1 g of chitosan was gradually added to 200 mL of 1% acetic acid with continuous stirring and heating on a magnetic stirrer at 350 rpm. Stirring continued until the solution was completely homogenized, after which propolis extract was added. The solution was then stirred for a further 15 min at room temperature.

In similar procedure, 1 g of glycerol was weighed into an Erlenmeyer flask, and 1% acetic acid was gradually added with gentle stirring on a magnetic stirrer. Then, 1 g of chitosan and propolis extract were gradually added. The solution was then stirred for a further 15 min at room temperature.

Both procedures are the first step in future modification of textile samples by propolis extract and by the investigated mixtures. In a future experiment we plan to pour pure propolis extract into a Teflon container placed on the base of the dip-coating apparatus. The cotton fabric sample is going to be modified so that it is immersed and withdrawn at a speed of 1 mm/s, resulting in a coating on both sides of the fabric. After modification, the samples will be left for 24 h at room temperature and then heated at 30 °C for additional hardening. Such samples are foreseen in medical applications.

The surface tension of the liquid samples was determined using the stalagmometer method. Surface tension was determined using a stalagmometric method (Kocour, Chicago, IL, USA), with water serving as the reference liquid under room temperature and standard laboratory conditions. The procedure involved counting the number of drops released from the stalagmometer for both the reference liquid and the test samples, allowing the surface tension to be calculated based on the ratio of drop numbers and known physical properties of water. All measurements were performed in triplicate to ensure reproducibility and minimize experimental error. The use of water, whose physical properties were determined at 20 °C and measured in the laboratory as surface tension of 72.75 mN/m and density of 0.99820 g cm^−3^, provided a reliable basis for comparison and accurate determination of the surface tension values of the examined solutions.

Table 4 presents the dependance of the surface tension on the number of drops recorded from the stalagmometric determination of surface tension.

Coffee ring preparation

Sample solutions containing active propolis compound such as morine were prepared at their maximum solubility (0.25 mg mL^−1^). Droplets were dispensed under tightly controlled conditions: 0.5 bar pressure, 25 ms dosing time, and 30 G needle positioned 0.5 mm above the substrate, at 22–24 °C and 40–50% relative humidity. Samples were dried undisturbed to preserve the integrity of the patterns. Optical microscopy was the primary tool for observing ring formation and analyzing ring shape, size, particle distribution, and morphology.

Optical microscopy (OM)

Coffee ring patterns were characterized using an Olympus BX53M optical microscope with polarization (Olympus, Breinigsville, PA, USA). Morphology, uniformity, and ring structure were examined to assess particle distribution and the effect of solvents.

## 4. Conclusions

The combined use of TLC, UV-VIS, and FTIR spectroscopy proved to be an effective and complementary strategy for the rapid characterization of raw propolis extract without pre-cleaning steps other than sonication that could remove trace-level bioactive compounds. One- and two-dimensional TLC enabled separation and preliminary profiling of propolis extract sample, with 2D-TLC showing 27% higher resolution and allowing detection of overlapping flavanones not observable in 1D-TLC. UV-VIS spectroscopy provided insight into the solubility behavior of propolis in various solvents, which was further supported by coffee ring experiments. FTIR analysis confirmed the presence of characteristic functional groups such as phenols, esters, and flavonoids, highlighting the chemical diversity of the sample. Together, these non-destructive techniques offer a reliable and reproducible framework for evaluating propolis composition, supporting both quality control and standardization. This multidimensional analytical approach is particularly valuable for the development of antimicrobial and antiviral propolis-based coatings, as it ensures the detection, preservation, and structural integrity of bioactive constituents crucial for biological efficacy.

## Figures and Tables

**Table 1:** R_F_ values of flavonoid and phenolic compounds detected in the investigated propolis extract sample. * Compounds detected only by 2DTLC.

Compound	R_F_ Value	Chemical Composition
Galangin	0.94	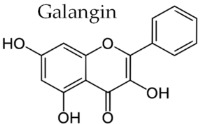
Chrysin	0.90	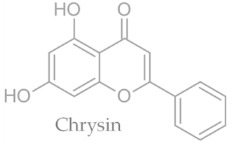
Naringenin	0.76	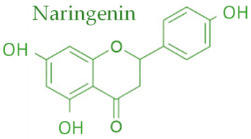
Ferulic acid	0.70	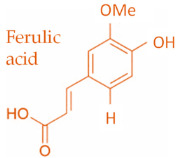
Caffeic acid	0.59	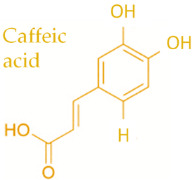
Myricetin	0.54	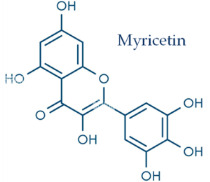
Flavanone *	0.50	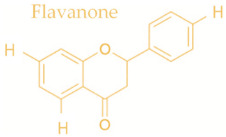
Flavone *	0.44	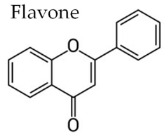
3-Hydroxyflavone *	0.13	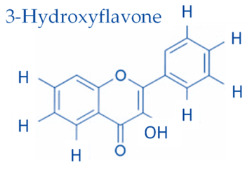
Gallic acid	0.08	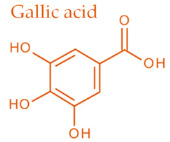
Chlorogenic acid	0.01	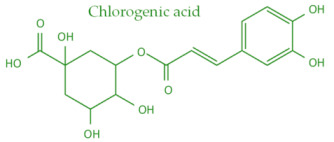

**Table 2:** R_F_ values of reference standards reported in the literature [39].

Standard	A *	B *	C *	D *	E *	Standard	A *	B *	C *	D *	E *
Flavanone	0.67	0.40	0.62	0.65	0.38	Morin	0.23	0.16	0.14	0.14	0.13
Naringenin	0.54	0.37	0.58	0.44	0.24	Chrysin	0.62	0.38	0.60	0.53	0.36
Flavone	0.88	0.62	0.92	0.86	0.66	Quercetin	0.39	0.27	0.27	0.28	0.22
3-Hydroxyflavone	0.77	0.51	0.80	0.76	0.56	Galangin	0.65	0.44	0.64	0.57	0.37
6-Hydroxyflavone	0.67	0.39	0.61	0.62	0.36	Apigenin	0.44	0.33	0.47	0.33	0.21
6′-Hydroxyflavone	0.52	0.32	0.46	0.51	0.28	Kaempferol	0.51	0.37	0.50	0.39	0.23
7-Hydroxyflavone	0.46	0.30	0.42	0.42	0.26	o-Coumaric acid	0.55	0.38	0.51	0.51	0.37
3,6-Dihydroxyflavone	0.54	0.36	0.51	0.52	0.34	Caffeic acid	0.38	0.26	0.30	0.33	0.22
3,7-Dihydroxyflavone	0.54	0.36	0.50	0.48	0.33	Ferulic acid	0.56	0.32	0.49	0.49	0.28

* The R_F_ values of flavonoids and phenolic acids reported by Medić Šarić et al. for the following TLC solvent systems (labeled as A to E): A consisted of toluene/ethyl acetate/formic acid in a 36:12:5 volume ratio; system B used cyclohexane/ethyl acetate/formic acid at 30:15:5; system C employed toluene/ethyl acetate/acetic acid at a 36:12:5 proportion, and system D consisted of cyclohexane/ethyl acetate/acetic acid at 31:14:5. Finally, system E was composed of n-hexane/ethyl acetate/formic acid in a 31:14:5 ratio. These solvent systems allowed for the separation and comparison of the migration patterns of the various flavonoid and phenolic acid standards under different polarity conditions. Moreover, detailed information on four other systems is reported and elaborated by Medić Šarić et al. [39].

**Table 3:** Solvents used in TLC experiments and their volume ratios.

Solvent Mixture	Volume Ratio of Solvents
Benzene—Methanol	95:5
Methanol—Acetic Acid	90:10
Toluene—Ethyl acetate—Formic acid	36:12:5
Cyclohexane—Ethyl acetate—Formic acid	30:15:5
Toluene—Ethyl acetate—Acetic acid	36:12:5
Cyclohexane—Ethyl acetate—Acetic acid	31:14:5
n-Hexane—Ethyl acetate—Formic acid	31:14:5
Toluene—Acetone—Formic acid	38:10:5
n-Hexane—Ethyl acetate—Acetic acid	31:14:5
Petroleum ether—Ethyl acetate—Formic acid	30:15:5

**Table 4:** Surface tension and the number of drops recorded from the stalagmometric investigation.

Solvent	Number of Drops	Standard Deviation	Calculated Surface Tension γ, mN/m
EtOH	31	1.50	83.45
H_2_O	32	1.11	88.83
Glycerine	30	0.52	80.76
Carboxymethyl cellulose	27	1.65	72.68
Chitosan	28	0.51	75.37
Chitosan/Glycerine	27	1.02	72.68
Chitosan/HAc	27	1.02	72.68

## Data Availability

Detailed information on the project and the supporting data is available at the web page: https://www.fkit.unizg.hr/CoRe4Pharm (accessed on 30 October 2025).
